# Artificial Intelligence Evidence-Based Current Status and Potential for Lower Limb Vascular Management

**DOI:** 10.3390/jpm11121280

**Published:** 2021-12-02

**Authors:** Xenia Butova, Sergey Shayakhmetov, Maxim Fedin, Igor Zolotukhin, Sergio Gianesini

**Affiliations:** 1 Department of Fundamental and Applied Research in Surgery, Pirogov Russian National Research Medical University, 117997 Moscow, Russia; ksuynec@mail.ru; 2 Department of Radiotechnics, Faculty of Technical Cybernetics, National Research University of Electronic Technology, 124498 Moscow, Russia; 2411777@gmail.com; 3 Department of Data Science, Faculty of Information Technology, Monash University, Melbourne 3800, Australia; m-fedin@mail.ru; 4 Department of Translational Medicine, University of Ferrara, 44121 Ferrara, Italy; sergiogianesini@gmail.com; 5 Department of Surgery, Uniformed Services University of Health Sciences, Bethesda, MD 20814, USA

**Keywords:** artificial intelligence, deep machine learning, peripheral artery disease, chronic venous disease, venous thromboembolism

## Abstract

Consultation prioritization is fundamental in optimal healthcare management and its performance can be helped by artificial intelligence (AI)-dedicated software and by digital medicine in general. The need for remote consultation has been demonstrated not only in the pandemic-induced lock-down but also in rurality conditions for which access to health centers is constantly limited. The term “AI” indicates the use of a computer to simulate human intellectual behavior with minimal human intervention. AI is based on a “machine learning” process or on an artificial neural network. AI provides accurate diagnostic algorithms and personalized treatments in many fields, including oncology, ophthalmology, traumatology, and dermatology. AI can help vascular specialists in diagnostics of peripheral artery disease, cerebrovascular disease, and deep vein thrombosis by analyzing contrast-enhanced magnetic resonance imaging or ultrasound data and in diagnostics of pulmonary embolism on multi-slice computed angiograms. Automatic methods based on AI may be applied to detect the presence and determine the clinical class of chronic venous disease. Nevertheless, data on using AI in this field are still scarce. In this narrative review, the authors discuss available data on AI implementation in arterial and venous disease diagnostics and care.

## 1. Introduction

The need for optimizing healthcare management by guaranteeing both top quality consultation and the rationalization of the available infrastructure resources is of paramount importance, particularly in a post COVID-19 pandemic world [1]. Not only might remote consultation be helpful in such situations as a pandemic-induced lock-down, but it may also be of use in rural areas with a limited access to well-equipped healthcare centers [2]. Diagnostic tools based on artificial intelligence (AI)-dedicated software and digital medicine in general are considered an effective solution for remote consultations [1,3].

The term “AI” is used to indicate a simulation of human intellectual behavior by a computer with minimal human intervention [4]. AI is based on a machine learning process or on an artificial neural network (ANN). Machine learning indicates a set of technologies that automatically detect patterns in data and then use them to predict future data or enable decision making under uncertain conditions. Deep learning is part of the machine learning process and is a special type of artificial neural network that resembles a system of synapses between human neurons. The ANN consists of many basic computing units, i.e., artificial neurons that use a simple classifier model. After weighing the evidence, every neuron of an ANN produces a decision signal. To train an ANN, learning algorithms, such as back propagation, are involved. Paired input signals and desired output decisions resemble brain functioning when it analyzes external sensory stimuli to perform different activities depending on the situation. However, no one knows how exactly AI-based tools make conclusions, as they operate like a “black box” [5,6,7,8].

Deep learning is particularly compelling due to its usage in processing and analyzing big data in healthcare, since deep learning can be used to find solutions for effective patient management. Using AI in everyday practice may provide accurate diagnostic algorithms and personalize patient management [9,10].

AI may reduce medical mistake rates and prevent discrepancies in diagnostic data interpretation [7]. Moreover, AI can be used to support cost-effective clinical decision-making, developing healthcare recommender systems, emotion recognition using physiological signals, and patient monitoring [3,11,12]. This can also significantly reduce the burden on healthcare workers, maximizing their time and expertise for optimal patient care [13].

AI has already been used in diagnostics for “object detection” (lesion localization), “object segmentation” (determination of the contours and boundaries of the lesion), and “classification of objects” (malignant or benign) [14,15]. This makes AI useful in radiology, where large datasets are processed [16]. AI interprets images with breast cancer, including lymph nodes metastases [17,18,19]. Analyzing chest radiographs with AI helps to detect lung cancer, metastases, tuberculosis or pneumonia, or diffuse lung diseases [20,21]. The automatic detection and segmentation of brain metastases, as well as prostate cancer detection on magnetic resonance (MRI) images, is another field for AI utilization [22,23]. In ophthalmology, AI helps to diagnose diabetic retinopathy, age-related macular degeneration, glaucoma, and other ophthalmic disorders [24,25,26,27]. In traumatology, neural networks in X-ray diagnostics of intertrochanteric fractures of the proximal femur surpassed conclusions of orthopedic surgeons. Ultra-precise neural networks have significant potential for fracture screening on plain radiographs when orthopedic surgeons are not available in emergency situations [28]. AI allows detection of early-stage melanoma on skin images [29]. Neural networks are as effective as certified dermatologists in differentiating benign neoplasms from malignant neoplasms on photographic and dermatoscopic images [30]. AI can be used to evaluate electrocardiograms [31] and ultrasound images [32], and in pathomorphology [33] and genomics [34]. AI is a promising tool that can be used to trace contacts during the pandemic, to improve pneumonia diagnostics [35], or to monitor COVID-19 patients [36].

Two of the possible areas where AI based diagnostics seems promising are in arterial atherosclerotic images and lower limb venous diseases. We performed a literature search using the following keywords: “artificial intelligence”, “deep machine learning”, “artificial neural network”, “convolutional neural network”, “telehealth”, “peripheral artery disease”, “abdominal aortic aneurism”, “deep venous thrombosis”, “pulmonary embolism” “venous thromboembolism”, “chronic venous disease”, “varicose veins”, “venous ulcer”, “vascular surgery”, “vascular medicine”, “angiology”, and “phlebology”. We then assessed all the articles for their eligibility. The reference list of all the articles related to AI in vascular management was searched for additional sources that contributed to the field.

The present narrative review reports the current state of the art of AI in the management of arterial disease, venous thromboembolism (VTE), and chronic venous disease (CVD).

## 2. Artificial Intelligence in Arterial Disease

AI is still not widely used in vascular medicine, so not that many publications can be found while searching the literature [37]. However, various AI algorithms are currently being developed in this field. Vascular segmentation is challenging because the vessels are highly variable in morphology, size, and curvature. Nevertheless, AI can help in segmentation and pattern recognition, therefore improving diagnostic efficiency and reducing time spent on analyzing data.

AI opens up many opportunities in vascular surgery, including the management and analysis of medical data, and the development of expert systems for prediction and decision making. It can also be used for patient care, in education and training of vascular surgeons, and as a health information and surveillance system for research [38].

Kurugol S. et al. presented a tool to assess aorta morphology and aortic calcium plaques on CT scans. The authors computed the agreement between the proposed algorithm and expert segmentations on 45 CT scans and obtained a closest point mean error of 0.62 ± 0.09 mm and a Dice coefficient of 0.92 ± 0.01 [39].

Graffy P.M. et al. used instance segmentation with convolutional neural networks (Mask R-CNN). It was applied to a dataset of 9914 non-contrast CT scans from 9032 consecutive asymptomatic adults who had undergone colonography screening. A developed fully automated abdominal aortic calcification scoring tool allows for the assessment of any non-contrast abdominal CT for cardiovascular risk [40].

AI can also help with segmentation analysis of ultrasound, CT, and magnetic resonance imaging (MRI) images in patients with carotid artery stenosis [41,42]. Caetano Dos Santos F.L. et al. developed a tool for the segmentation and analysis of atherosclerosis in the extracranial carotid arteries. A dataset of 59 randomly chosen head-and-neck CTA scans was used. An algorithm mainly based on the detection of carotid arteries, delineation of the vascular wall, and extraction of the atherosclerotic plaque was successful in 83% of stenoses over 50%. Specificity and sensitivity were 25% and 83%, respectively, with an overall accuracy of 71% [43].

Raffort J. et al., while searching the literature on AI tools for abdominal aortic aneurism management, found several prognostic programs. The potential of AI was confirmed in predicting aneurism growth and rupture, in-hospital and 30 day mortality, endograft complications, aneurism evolution, stent graft deployment, and the need for re-intervention after endovascular aneurysm repair. Nevertheless, small datasets were mainly used, while machine learning approaches require large databases for learning and training. A lack of external validation is also a pitfall as it outlines the need for multicenter registries [44].

Dehmeshki J. et al. presented a computer-aided detection tool that detected arteries based on a 3D region growing method and a fast 3D morphology operation. They developed a computer-aided measurement system to measure the artery diameters from the detected vessel centerline. The system has been tested on phantom data and on fifteen CTA datasets of peripheral arterial diseases (PAD) patients. An 88% detection accuracy of stenosis was achieved with an 8% error of measurement accuracy [45].

Ross E.G. et al. used machine learning algorithms to analyze electronic health records. Data of 7686 PAD patients from two tertiary hospitals were used to learn predictive models. Models confirmed the ability of machine learning algorithms to identify patients with peripheral arterial atherosclerosis, favoring an early detection of the subjects at risk of serious cardiac and cerebrovascular events [46].

AI was confirmed as an extremely useful tool for teaching goals by simulating different clinical cases. Virtual reality simulators are already available to train young professionals in basic endovascular skills [47,48].

AI is used in apps designed for use by patients themselves. Those applications analyze photographic images of the legs and help to identify early signs of diabetic foot syndrome [49]. Ohura N. et al. prepared four architectures to build wound segmentation convolutional neural networks (CNNs). The best results were shown by U-Net, which demonstrated an area under the curve of 0.997, specificity of 0.943, and sensitivity of 0.993. Such tools may be applied to diagnostics of arterial and venous leg ulcers as well as pressure ulcers [50].

## 3. Artificial Intelligence in Venous Thromboembolism

Clinical decisions in patients with pulmonary embolism (PE) can be based on AI-supported analysis of multi-slice computed tomography (MSCT-angiography) of pulmonary arteries images [51,52]. For those patients, timely diagnostics are crucial to save lives, but in routine practice, PE is still one of the most commonly missed diagnoses [53]. Deep machine learning to detect PE on MSCT angiograms was demonstrated to be a valuable solution [54].

The first attempts to detect PE using neural networks were made in the early 1990s. Patil S. et al. supposed that computerized pattern recognition could accurately estimate the probability of PE based on readily available clinical characteristics. Medical history data, physical examination, ECG, chest X-ray scans, and arterial blood gases of patients with suspected acute PE were downloaded to a back propagation neural network. Study data were obtained from 1213 patients in a prospective study on PE diagnostics. They were divided into training group A (n = 606) and test group B (n = 607). These groups were then transformed into training set B (n = 607) and test set A (n = 606). The performance curve was constructed from clinical assessments made by specialists and the neural network in groups A and B. The areas under the corresponding ROC curves were 0.7450, 0.7477, and 0.7324. All differences were not significant. Thus, neural networks were able to predict the clinical probability of PE with an accuracy comparable to that of experienced clinicians [54].

Huang S.C. et al. used a deep learning model capable of detecting PE signs on computed tomography pulmonary angiography (CTPA) images of the pulmonary arteries with simultaneous data interpretation (77-layer 3D convolutional neural network). Researchers conducted a retrospective data collection of 1797 images from 1773 patients. Training (1461 images from 1414 patients), validation (167 images from 162 patients), and hold-out test sets (169 images from 163 patients) were developed. Stratified random sampling was used to create validation and test kits to ensure an equal number of positive and negative cases. There was no patient overlap between sets. When tested, the deep learning model achieved an AUROC score of 0.84 with automatic detection of PE signs on the test set. Thus, the possibility of using deep learning to evaluate complex radiographic data of CTPA angiograms to detect PE was confirmed [55].

Nima Tajbakhsh et al. presented a computer-aided detection of PE on CTPA images. They assumed that despite acceptable sensitivity, existing computer detection systems generate a large number of false-positive results. This may lead to additional burden on radiologists to analyze them. The possibility of convolutional neural networks (the type of neuronets specified for image classification) to eliminate false conclusions was investigated. Developing the “correct” representation of the image is important for the accuracy of AI when analyzing an object in 3D images. For this purpose, a multi-plane image of emboli with alignment along the vessels was developed. This imaging provides three advantages: (1) compactness, i.e., concise summarization of 3D contextual information around the embolus in two image channels; (2) consistency—automatic alignment of the embolus on two-channel images according to the orientation of the affected vessel; and (3) extensibility—natural support for data augmentation for training neural networks. This method was tested using a set of 121 CTPA angiograms with a total of 326 emboli. The sensitivity reached 83% with two false-positive results [56].

The important role of AI-based tools in the recognition of PE signs on CTPA images was also confirmed by other researchers [57,58,59,60].

Deep learning is used in cancer patients who are at high risk of VTE [61]. Randomized studies have shown that prophylactic doses of anticoagulants reduce VTE rates in cancer patients by about half [62]. However, there is also a potentially high risk of bleeding associated with anticoagulant therapy [63]. The decision to use anticoagulants for the prevention of cancer-related VTE should ideally be based on an effective risk stratification strategy [64].

Pabinger I. et al., from the University of Vienna, have developed and tested a computer-based predictive model of VTE based on AI in outpatients with cancer. They used data from 1737 patients from a CATS study (a prospective single-center observational cohort with a baseline biobank) who had recently been diagnosed with active cancer or disease progression after complete or partial remission. Patients who had a malignancy, except primary brain tumors, or lymphoma were selected. Only tumor localization, which is the most important part of the Khorana score, and D-dimer were left for training the model [65].

To test the model’s performance, demographic, laboratory data, and data from a multinational cohort study were used to identify cancer patients at high risk of VTE [66]. With the threshold of predictable cumulative 6 month’s risk of PE set at 10%, model sensitivity was 33% (95% confidence interval (CI) 23–47) and specificity was 84% (95% CI 83–87). The positive predictive value was 12% (95% CI 8–16), and the negative predictive value was 95% (95% CI 94–96). With the threshold set at 15%, model sensitivity was 15% (95% CI 8–24) and specificity was 96% (95% CI 95–97). The positive predictive value was 18% (95% CI 9–29), and the negative predictive value was 95% (95% CI 94–96). This shows that the model may help to find patients eligible for pharmacological thromboprophylaxis [65,67].

Huang C. et al. conducted a study using a fully automatic method of determining DVT extension. AI was used to detect the proximal level of deep vein thrombosis (DVT) on contrast-enhanced MRI images. Images taken from 58 patients with recently diagnosed lower limb DVT were analyzed. A total of 5388 snapshots were made, and on 2683 of them, thrombotic masses were seen. The boundaries of the blood clots on the CT scan were manually delineated by radiologists, and then a deep learning-based neural network was trained. The basic principle of operation is based on the segmentation of the boundaries of thrombosis. A DL network with an encoder–decoder architecture was designed for DVT segmentation. It took about 1.5 s for this model to fulfill the task and to identify the thrombus extension. This model identifies vein segments with thrombosis on the MRI image. The average value of the Dice similarity coefficient (DSC) for 58 patients was 0.74 ± 0.17, and the average value of the DSC was 0.79 (range 0 ~ 0.91). The results showed that the proposed method is relatively effective and quick. If further improved, this method will help clinicians to evaluate DVT quickly and objectively [68].

Willan J et al. confirmed that AI may be applied for risk stratification in patients with suspected DVT [69]. A neural network was trained to stratify the probability of DVT in patients with suspected DVT using the data of the 11,490 consecutive cases, including 7080 cases for which all data, i.e., Wells score, D-dimer, and duplex ultrasound, were available. The network was able to exclude DVT without the necessity for ultrasound scanning in more patients as compared with the existing algorithm with low false-negative rates. After preliminary fast and reliable AI evaluation, patients with symptoms suggestive of DVT can then be sent to the hospital for vascular specialist examination in order to confirm or exclude thrombosis [70]. To do this, the Wells scale has been used to assess symptoms, therefore identifying patients with high DVT probability [71]. AI can become a powerful synergistic tool, together with D-dimer assessment, for better prioritization of the ultrasound scanning and consequent disease management [72,73,74,75].

Deso S. et al. developed CNN to detect 23 different types of inferior vena cava (IVC) filters and diagnostics of related complications. For each type of cava filter, a database of radiographs and CT scans was collected. A wireframe and storyboard were created, and software was developed using HTML5/CSS compliant code [76].

Ni J.C. et al. used deep learning for automated classification of IVC filter types on radiographs. They took 1375 cropped radiographic images of 14 types of IVC filters, with 139 images for a test set. The CNN classification model achieved an F1 score of 0.97 (0.92–0.99) for the test set overall and of 1.00 for 10 of 14 individual filter types. Of the 139 test set images, 4 (2.9%) were misidentified, all mistaken for other filter types that appeared highly similar [77].

## 4. Artificial Intelligence in Chronic Venous Disease

CVD is a highly prevalent disease [78], severely impacting quality of life and the national health care budget [79]. Being a chronic condition, CVD needs a permanent follow-up, which is not easy for many patients, especially in the rural regions where access to vascular care is limited in many geographical areas. For rural residents, AI-supported diagnostics seems to be a good option [80,81]. Automatic methods based on AI may be applied to detect the presence and determine the clinical class of CVD. Nevertheless, data on using AI in this field are scarce.

Fukaya E. et al. used machine learning to find genetic risk factors for varicose veins in 493,519 people at the British Biobank. In addition, a genome-wide study of the association of varicose veins was carried out among 337,536 people, followed by a quantitative analysis of loci and expression pathways. They used a gradient boosting machine model. Its principle of operation is based on the introduction of variable data, analysis, and construction of a new tree for predicting possible options. The relationship of the genotype with the presence of varicose veins was tested using a logistic model. Researchers have found that high growth persons are at a higher risk of varicose vein development [82].

Another promising area for AI is varicose vein recurrence risk estimation after invasive procedures. Bouharati I. et al. analyzed risk factors for recurrence, such as age, sex, obesity, genetic predisposition, inadequate diagnosis, double trunk of the great saphenous vein, double trunk of the small saphenous vein, neovascularization, technical failures, and time from procedure. A CNN system was constructed with probable causes of varicose recurrence as input variables and recurrence rate as the output variable. To train neural networks, data on 62 patients who had undergone invasive treatment were used, but no results of how the system performed were published [83].

Artificial neural networks may predict the healing time for venous ulcers [84], therefore helping health professionals in customizing treatment and, at best, improving the patient’s quality of life [85,86,87]. Taylor R. J. et al. retrospectively assessed data on 325 patients with 345 venous ulcers. An ANN based on a computer program (a simple neural network) was used for training. It was loaded with input data on 45 risk factors and ulcer healing time as output. After training, the ANN accurately predicted the healing time in 68% of cases. AI also identified the most important risk factors for ulcer healing. Among them are previous history of venous ulcers, profuse ulcerative exudate, high body mass index, large initial surface skin defect, age, and male sex. The neural network confirmed its ability to predict which ulcer may be resistant to standardized treatment [84].

Bhavani R. et al. used AI to determine venous ulcer stages on photo images. They obtained data from 150 patients. From each patient, 5–15 photographs were taken, with a total of 1770 images for training and 810 for testing. The scheme of the neural network operation consisted of four parts. Images were previously edited to remove flash light reflection. Then, contour segmentation of the ulcer surface was carried out, and the analysis was performed using a multidimensional ultra-precise neural network. During the extraction stage, features such as homogeneity, color, texture, and depth, were analyzed. This tool had an average accuracy of 99.55%, with specificity of 98.06% and sensitivity of 95.66% [88,89].

The most burdensome venous pathology is primary varicose veins, which affect 27–31% of the general population in rural settlements [90]. To diagnose and manage varicose veins in residents of distant areas, AI-based applications seem to be good tools. To date, few studies have been published on the assessment of AI automatic methods for varicose vein detection.

Qiang Shi and co-authors used 221 photographic images of the lower limbs in order to train the neural network to identify CVD classes according to CEAP classification. They mapped low-level image features onto middle-level semantic features using a concept classifier. Then, a multi-scale semantic model was created. The latter model was used to represent images with rich semantics. Finally, a scene classifier was trained using an optimized feature subset and was then used to determine CVD clinical class. The reported accuracy was 90.92% [91].

Hoobi M.M. et al. conducted a similar study based on the analysis of only 100 photographic images (60 with varicose veins, 40 with no CVD). The system used more than one type of distances with a probabilistic neural network to produce a diagnostic system of CVD with high accuracy. New pictures were used when testing 60 of the images. In this CNN model, shape, size, and texture of the skin with varicose veins were used with a reported accuracy of 94% [92].

Most vascular AI tools are based on neural networks trained with a limited number of images (Table 1). It is generally considered that 1000 cases are needed just to build a system, while if aiming for practical use, the number of images for training has to be closer to 100,000 [14]. This is especially true for CVD, which is classified for seven clinical classes, among which varicose veins represent only one of them. Moreover, even in large training samples, the recognition result strongly depends on the conditions of photo taking, including the image resolution, the relative size of the lesion to the total area of the image, the position of the leg, and the degree of the hair line.

## 5. Conclusions

AI has demonstrated to be an extremely helpful tool in healthcare, particularly in a time in which healthcare resources must be optimized, such as during a pandemic and in the rural conditions. Application of AI in healthcare has the potential to bring financial benefits and savings. However, according to this literature search, these services need further validation before they are used routinely in clinical practice, making this topic of great interest for the healthcare community. This is particularly true for CVD as a condition highly impacting society and because of its healthcare costs.

## Figures and Tables

**Table 1:** AI tools based on learning with images.

Authors, Year	Disease	AI Used for	Data Used for AI Learning	Principle of Operation	Number of Images	Performance Metrics	App Available Online
Kurugol S. et al., 2015 [39]	PAD	Aorta size calculation, morphology, mural calcification distributions	CT images	Convolutional neural networks (Mask R-CNN)	2500	Dice coefficient of 0.92 ± 0.01	No
Caetano Dos Santos F.L. et al., 2015 [43]	Carotid arteries stenosis	Segmentation and analysis of atherosclerotic lesions in extracranial carotid arteries	CTA images	Convolutional neural networks	59	71% accuracy	Yes
Raffort J. et al., 2015 [44]	Abdominal aortic aneurism (AAA)	Quantitative analysis and characterization of AAA morphology, geometry, and fluid dynamics	CT images	Convolutional neural networks	40	93% accuracy	No
Dehmeshki J. et al., (2014) [45]	PAD	Arterial network, artery centerline detection, and distortion correction	CTA images	Computer-aided detection system	15	88% accuracy	No
Huang SC. et al., (2020) [55]	VTE	PE detection	CTPA images	Convolutional neural network	1797	AUROC score of 0.84	No
Huang C. et al., 2019 [68]	DVT	Proximal level of DVT detection	Contrast-enhanced MRI images	Convolutional neural network	5388	Dice coefficient of 0.79	No
Ni J.C. et al., 2020 [77]	DVT	Different inferior vena cava filters identification	Radiographic images	Deep-learning convolutional neural network	1375	F1 score of 0.97	Yes
Rajathi V., Bhavani R.R., Wiselin Jiji G. (2019) [88]	CVD	Venous ulcer detection	Venous ulcers photos	Region growing, K-means, kNN	1770	94.85% accuracy	No
Shi Q., et al., 2018, [91]	CVD	Varicose vein detection	Lower limbs photos	Multi-scale semantic model constructed to form the image representation with rich semantics	221	90.92% accuracy	No
Hoobi M.M., Qaswaa A., 2017, [92]	CVD	Varicose vein detection	Lower limbs photos	Probabilistic neural network	100	94% accuracy	No
